# Modeling Signal Transduction Leading to Synaptic Plasticity: Evaluation and Comparison of Five Models

**DOI:** 10.1155/2011/797250

**Published:** 2011-02-22

**Authors:** Tiina Manninen, Katri Hituri, Eeva Toivari, Marja-Leena Linne

**Affiliations:** 1 Department of Signal Processing, Tampere University of Technology, P.O. Box 553, 33101 Tampere, Finland

## Abstract

An essential phenomenon of the functional brain is synaptic plasticity which is associated with changes in the strength of synapses between neurons. These changes are affected by both extracellular and intracellular mechanisms. For example, intracellular phosphorylation-dephosphorylation cycles have been shown to possess a special role in synaptic plasticity. We, here, provide the first computational comparison of models for synaptic plasticity by evaluating five models describing postsynaptic signal transduction networks. Our simulation results show that some of the models change their behavior completely due to varying total concentrations of protein kinase and phosphatase. Furthermore, the responses of the models vary when models are compared to each other. Based on our study, we conclude that there is a need for a general setup to objectively compare the models and an urgent demand for the minimum criteria that a computational model for synaptic plasticity needs to meet.

## 1. Introduction

Neurons respond to variations in extracellular and intracellular environment by modifying their synaptic and intrinsic membrane properties. When a presynaptic neuron passes an electrical or chemical signal to a postsynaptic neuron, changes in the synapse occur. Long-term potentiation (LTP), also known as strengthening, and long-term depression (LTD), also known as weakening, of synapses are two forms of synaptic plasticity. Both LTP and LTD participate in storing information and inducing processes that are thought to ultimately lead to learning (see, e.g., [1]). The main focus in the research on synaptic plasticity in vertebrates has been on LTP and LTD in cornu ammonis 1 (CA1) region of the hippocampus [1] because hippocampus is especially important in the formation and retrieval of declarative memories. Several mechanisms have been shown to be the reason for changes in synaptic strength; for example, changes in neurotransmitter release, conductivity of receptors, numbers of receptors, numbers of active synapses, and structure of synapses [2].

At present, there are more than a hundred molecules found important in LTP/LTD, some of which are key components for LTP/LTD formation and others being able to modulate the ability to generate LTP/LTD [1]. Strong evidence supports the finding that calcium (Ca^2+^)/calmodulin (CaM)-dependent protein kinase II (CaMKII) meets the criteria for being the essential molecule to LTP [3]. Protein kinases add phosphates to proteins, and, on the other hand, protein phosphatases remove phosphates from proteins to activate or deactivate them. It is hence straightforward to consider that also the protein phosphatases, such as protein phosphatases 1, 2A, and 2B (PP1, PP2A, and PP2B, a.k.a. calcineurin (CaN)), have important roles in synaptic plasticity [4].

More than a hundred computational models, simple and more complex ones, have been developed to describe the mechanisms behind synaptic plasticity at the biochemical level (see, e.g., [5, 6]). Simplest models only have one reversible reaction (see, e.g., [7]) and most complicated ones several hundred reactions (see, e.g., [2]). The communities of researchers in computational systems biology and neuroscience are in a need for a general setup on how to evaluate and classify the models for synaptic plasticity (see also [5]). Because the statistical data from the models does not necessarily represent exactly the same phenomenon, mathematical methods, such as Bayesian methods [8–10], are not applicable to comparison of these synaptic plasticity models. Thus, some subjective selection of features describing the overall behavior of the modeled system and traditional simulation-based comparison are required. To enable the use of previous computational models for synaptic plasticity, minimum criteria for the models need to be set (see BioModels projects, e.g., [11, 12]).

The aim of this study is to provide the first comparison of synaptic plasticity models by computational means and to be the first step towards finding a general setup for comparison. The organization of this study is as follows. First, we shortly describe the biology behind synaptic plasticity by presenting five computational models selected for this evaluation. Second, the used simulation setups, including the second messenger Ca^2+^ and neurotransmitter dopamine (DA) inputs, as well as the total concentrations of protein kinase CaMKII and protein phosphatase PP1, are presented. Third, we show the comparative simulation results and evaluate the synaptic plasticity models. The comparison is made between the two models selected for the same neuron type, that is, between the two models for a hippocampal CA1 neuron and between the two models for a striatal medium spiny neuron. We also examine if a generic model is suitable for describing the behavior of either of the two neuron types and thus being a good computational representative of them. Lastly, we discuss our most important findings and provide some conclusions.

## 2. Models and Methods

### 2.1. Biological Background

Several types of LTP and LTD can occur in the brain depending on the neuron type and given input to the neuron. LTP can be divided into two main types: an early phase LTP (E-LTP), which lasts for 1 h-2 h, and a late phase LTP (L-LTP), which persists for several hours [1, 3]. Similar division can also be made for LTD. All types of plasticity involve three processes: induction, expression, and maintenance. The LTP/LTD phenomenon can be induced by introducing glutamatergic and dopaminergic inputs. Glutamatergic input causes the elevation of intracellular Ca^2+^ concentration in postsynaptic density, meaning a small volume linking postsynaptic membrane receptors, their signaling pathways, and the cytoskeleton, and in cytosol. Dopaminergic input activates the enzyme adenylyl cyclase (AC) on the cell membrane and thus increases the intracellular cyclic adenosine monophosphate (cAMP) concentration. This input can only be found in some neuron types, for example, in striatal medium spiny neurons. Ca^2+^ and cAMP serve as secondary messengers passing the glutamatergic and dopaminergic signals forward and activating downstream proteins. In this study, the elevations in Ca^2+^ and DA concentrations are used as model inputs (see details in Section 2.3).

**Figure 1:**
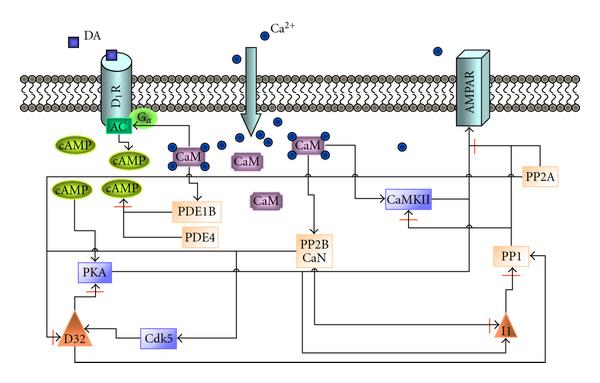
**Schematic representation of the postsynaptic mechanisms involved in signal transduction related to induction of LTP/LTD**. Intracellular calcium ions (Ca^2+^) bind to calmodulin (CaM), which further affects the activation of protein phosphatase 2B (PP2B) a.k.a. calcineurin (CaN), CaM-dependent kinase II (CaMKII), adenylyl cyclase (AC, the catalyst of the reaction producing cyclic adenosine monophosphate (cAMP)), and phosphodiesterase type 1B (PDE1B). Dopamine (DA) increases cAMP concentration via AC activation. Together with PDE1B, also PDE type 4 (PDE4) degrades cAMP. cAMP-dependent protein kinase (PKA) phosphorylates *α*-amino-3-hydroxy-5-methylisoxazole-4-propionic acid receptor (AMPAR) and protein phosphatase 1 (PP1) inhibitor 1 (I1). In addition, protein phosphatase 2A (PP2A) and cyclin-dependent kinase 5 (Cdk5) affect PP1 regulatory subunit a.k.a. DA- and cAMP-regulated neuronal phosphoprotein of 32 kDa (D32).

Briefly, the signal transduction network leading to LTP/LTD phenomenon includes the following events (see Figure 1). Elevated Ca^2+^ concentration enables the binding of Ca^2+^ to CaM which further activates CaM-dependent kinase CaMKII. Then Ca^2+^/CaM-CaMKII complex is able to proceed to autophosphorylation. Ca^2+^/CaM also binds to protein phosphatase CaN. The effect of active CaN on protein phosphatase PP1 activity is bidirectional; CaN inhibits PP1 inhibitor 1 (I1) and activates cyclic-dependent kinase 5 (Cdk5). Both of these actions lead to activation of PP1. However, active CaN is also able to deactivate PP1 regulatory subunit a.k.a. DA- and cAMP-regulated neuronal phosphoprotein of 32 kDa (DARPP32, D32 in Figure 1), which leads to deactivation of PP1. Active PP1 has a major role in dephosphorylating CaMKII and *α*-amino-3-hydroxy-5-methylisoxazole-4-propionic acid receptor (AMPAR). On the other hand, due to the DA input, cAMP activates cAMP-dependent protein kinase (PKA) which phosphorylates AMPAR (see synaptic plasticity mechanisms, e.g., in [1, 4]). In the ultimate end of the signaling cascade described in this study, protein kinases CaMKII and PKA, together with protein phosphatases PP1 and PP2A, act on AMPAR.

The phosphorylation and dephosphorylation of AMPAR subunits are crucial for the trafficking of AMPARs. Regulated AMPAR trafficking between intracellular, synaptic, and nonsynaptic membranes at the postsynaptic hippocampal neuron is found to provide a protein-level basis for controlling the amount of AMPARs on the plasma membrane and hence postsynaptic responsiveness [13, 14]. It is suggested that in the basal conditions, AMPARs are concentrated on the postsynaptic membrane but also exist abundantly in endosomal compartments, meaning the membranes inside the cell [15]. Some of the AMPAR subunits undergo constant recycling with membrane receptors in an activity-independent manner. However, the amount of AMPARs in the postsynaptic membrane shows only modest variation. Following the N-methyl-D-aspartate receptor (NMDAR) stimulation and CaMKII activation, exocytosis of AMPAR subunits from endosomal compartments to cell membrane is triggered, leading finally to the insertion of AMPARs into synapses [13]. On the contrary, in synaptic depression endocytotic mechanisms are activated and subunits of AMPARs are stored in endosomal compartments or degraded [13].

### 2.2. Selection of Models

We set our criteria for model selection to be the following: (1) the model for synaptic plasticity has to include adequate postsynaptic reactions and kinetics, (2) the model can be found in a database, (3) the model describes synaptic plasticity either in a hippocampal CA1 neuron or in a striatal medium spiny neuron, (4) the model uses Ca^2+^ as input, and (5) CaMKII and PP1 are included in the model.

We select the following models describing synaptic plasticity in a hippocampal CA1 neuron: 

(i) model by d'Alcantara et al. [16], 

(ii) model by Kim et al. [17]. 

In addition, we select the following models describing synaptic plasticity in a striatal medium spiny neuron: 

(i) model by Lindskog et al. [18], 

(ii) model by Nakano et al. [19]. 

Furthermore, we select one generic neuron model which is compared to models above: 

(i) model by Hayer and Bhalla [2]. 

The characteristics and components of the selected models are tabulated in Tables 1 and 2 (see also [5]). In total, several protein kinases (CaMKII, Cdk5, and PKA) and protein phosphatases (CaN, PP1, and PP2A) are included in the models. The models have similar elements and are in some cases directly based on each other. Kim et al. [17] take the model by Lindskog et al. [18] as their base. This might be confusing since the models are made for neurons in different brain areas, but, on the other hand, they share similar pathways. Furthermore, the model by Kim et al. [17] takes into account the G protein-linked PKA activation. Within the models describing synaptic plasticity in a striatal medium spiny neuron, Nakano et al. [19] take some of the reactions from the earlier model by Lindskog et al. [18] and then use similar AMPAR trafficking model as the generic model by Hayer and Bhalla [2]. These selected models are also partly based on other published models, but we list here just how these selected models are based on each other. It should be noted that the models selected for this study as such can be considered as advanced models in the computational neuroscience community.

**Table 1:** *Characteristics of models*. Tabulated characteristics are the simulation environment and integration method, phases of long-term potentiation and long-term depression, model inputs, model outputs chosen for this study, and size of the model based on the number of different chemical species or other model variables. Used abbreviations are *α*-amino-3-hydroxy-5-methylisoxazole-4-propionic acid receptor (AMPAR), calcium ion (Ca^2+^), Ca^2+^/calmodulin-dependent protein kinase II (CaMKII), cyclic adenosine monophosphate (cAMP), dopamine (DA), DA- and cAMP-regulated neuronal phosphoprotein of 32 kDa (DARPP32), early phase LTP (E-LTP), induction (Ind.), Ca^2+^ influx via NMDARs (), late phase LTP (L-LTP), long-term depression (LTD), long-term potentiation (LTP), N-methyl-D-aspartate receptor (NMDAR), and cAMP-dependent protein kinase (PKA).

Model	Simulation environment	Phases	Inputs	Outputs	Size
d'Alcantara et al. [16]	MATLAB, ode23 (explicit Runge-Kutta)	Ind. LTP/LTD	Ca^2+^	AMPAR	14
Kim et al. [17]	XPPAUT, adaptive stiff integration method	Ind. L-LTP	Ca^2+^, DA	CaMKII/PKA	49
Lindskog et al. [18]	XPPAUT, adaptive stiff integration method	Ind. E-LTP	Ca^2+^, DA	DARPP32	89
Nakano et al. [19]	GENESIS/Kinetikit, exponential Euler	Ind. LTP/LTD	Ca^2+^, DA	AMPAR	111
Hayer and Bhalla [2]	MATLAB, ode23s (based on Rosenbrock)	LTP/LTD	Ca^2+^, cAMP,	AMPAR	258

**Table 2:** *Model components*. Tabulated characteristics are the compartments, receptors, Ca^2+^ mechanisms, and signaling pathways modeled. Used abbreviations are adenylyl cyclase (AC), *α*-amino-3-hydroxy-5-methylisoxazole-4-propionic acid receptor (AMPAR), calmodulin (CaM), calcium/CaM-dependent protein kinase II (CaMKII), calcineurin (CaN), cyclin-dependent kinase 5 (Cdk5), dopamine receptor (D_1_R), dopamine- and cyclic adenosine monophosphate-regulated neuronal phosphoprotein of 32 kDa (DARPP32), inhibitor 1 (I1), phosphodiesterase type 1 (PDE1), PDE type 1B (PDE1B), PDE type 2 (PDE2), PDE type 4 (PDE4), cyclic adenosine monophosphate-dependent protein kinase (PKA), protein phosphatase 1 (PP1), and protein phosphatase 2A (PP2A).

Model	Compartments	Receptors	Ca^2+^ mechanisms	Signaling pathways
d'Alcantara et al. [16]	1 postsynaptic	AMPAR	CaM buffer	CaM, CaMKII, CaN, I1, PP1
Kim et al. [17]	1 spine	D_1_R	CaM buffer	CaM, CaMKII, CaN, G protein, I1, PDE1B, PDE4, PKA, PP1
Lindskog et al. [18]	1 spine	D_1_R	CaM buffer	AC, CaM, CaMKII, CaN, DARPP32, PDE1, PDE4, PKA, PP1, PP2A
Nakano et al. [19]	1 spine	AMPAR, D_1_R	CaM buffer	AC, CaM, CaMKII, CaN, Cdk5, DARPP32, I1, PDE1, PDE2, PKA, PP1, PP2A
Hayer and Bhalla [2]	1 dendritic, 1 postsynaptic, 1 spine-head	AMPAR	CaM buffer, 1-D diffusion of some of the molecules	AC, CaM, CaMKII, CaN, PKA, PP1

### 2.3. Simulation Setup

**Figure 2:**
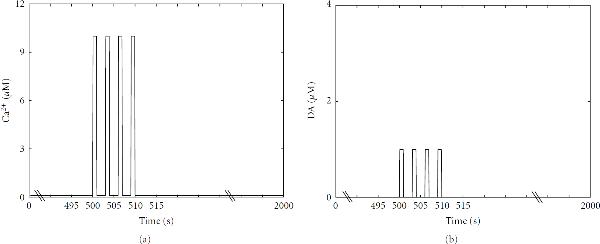
**Four-train (a) calcium (Ca ^2+^ ) and (b) dopamine (DA) inputs used in simulations**. 10 *μ*M Ca^2+^ and 1 *μ*M DA pulses are given for 1 s at time points , 503, 506, and 509 s. The duration of the basal plateau phases is thus 2 s. Before, between, and after the pulses a basal concentration of 0.1 *μ*M for Ca^2+^ and 0.01 *μ*M for DA is used.

For all the models, the total simulation time is 2000 s and a four-train Ca^2+^ input is given at  s in which the basal concentration of Ca^2+^ is 0.1 *μ*M and the pulse peak is 10 *μ*M (see Figure 2(a)). A four-train DA input (see Figure 2(b)), in addition to Ca^2+^ input, is given in the models that also model DA-related pathways, in other words to the models by Kim et al. [17], Lindskog et al. [18], and Nakano et al. [19]. Hayer and Bhalla [2] also use other inputs in addition to Ca^2+^ (see Table 1), and these other inputs are used similarly as presented in the original model. 

Six simulations (Sim1–Sim6) with different total concentrations of CaMKII and PP1 are run for all the models with the same inputs (see Table 3). These total concentrations are selected based on the different values used in the original models. Otherwise, we use the parameter values and mostly the initial concentrations given in the original models. In Table 4, we list the actual values that have to be changed to reach the simulation conditions given in Table 3.

**Table 3:** Total concentrations of CaMKII and PP1 ([CaMKII]_tot_, [PP1]_tot_) and ratios of them used in different simulations.

Sim ID	(*μ*M)	(*μ*M)	Ratio
Sim1	0.5	2	0.25
Sim2	1	4	0.25
Sim3	2	4	0.5
Sim4	4	1	4
Sim5	20	5	4
Sim6	20	2	10

**Table 4:** *Changed initial and total concentrations related to different states of CaMKII and PP1 to reach the total concentrations given in Table 3 .* Other values used in the simulations are based on the original models. We use here the actual names of the variables and constants as given in the model code downloaded from a database. Values are given in units of *μ*M.

Model	Sim1	Sim2	Sim3	Sim4	Sim5	Sim6
d'Alcantara et al. [16]	Naive states set to total, others zero	Naive states set to total, others zero	Naive states set to total, others zero	Naive states set to total, others zero	Naive states set to total, others zero	Naive states set to total, others zero

Kim et al. [17]	CK_ini = 0.5, , CKCaM = 0.01, CKpCaM = 0.01	CK_ini = 1, , CKCaM = 0.01, CKpCaM = 0.01	CK_ini = 2,	CK_ini = 4,	CK_ini = 20,	CK_ini = 20,

Lindskog et al. [18]	camkmax = 0.5,	camkmax = 1,	camkmax = 2,	camkmax = 4,	camkmax = 20,	camkmax = 20,

Nakano et al. [19]	CaMKII = 0.12, PP1_active = 0.87, PP1_I1_p = 0.60	CaMKII = 0.62, PP1_active = 1.87, PP1_I1_p = 1.60	CaMKII = 1.62, PP1_active = 1.87, PP1_I1_p = 1.60	CaMKII = 3.62, PP1_active = 0.29, PP1_I1_p = 0.18	CaMKII = 19.62, PP1_active = 2.37, PP1_I1_p = 2.10	CaMKII = 19.62, PP1_active = 0.87, PP1_I1_p = 0.60

Hayer and Bhalla [2]	basal_CaMKII_ PSD = 0.5, PP1-active_PSD = 2	basal_CaMKII_ PSD = 1, PP1-active_PSD = 4	basal_CaMKII_ PSD = 2, PP1-active_PSD = 4	basal_CaMKII_ PSD = 4, PP1-active_PSD = 1	basal_CaMKII_ PSD = 20, PP1-active_PSD = 5	basal_CaMKII_ PSD = 20, PP1-active_PSD = 2

It is assumed that the original models have been tested against changes in the values of parameters and initial concentrations, and thus no detailed sensitivity analysis is performed in this study. It is beyond the scope of this study.

We want to emphasize that the purpose of this study is not to perform any detailed analysis of the used integration methods nor to implement the models using other integration methods. Instead, we use the model as it is presented in the model database and simulate it using the given simulation tool. 

## 3. Results

### 3.1. Simulation Results

We evaluate and compare different computational models describing LTP and LTD phenomena based on the model outcomes. The comparison is made between the two models selected for the same neuron type; that is, two models are compared for a hippocampal CA1 neuron [16, 17] and two models for a striatal medium spiny neuron [18, 19]. In addition, we examine if a generic model [2] is a suitable approximation for hippocampal and striatal neurons in terms of reproducing the main LTP phenomenon. The model selection is justified upon the importance of AMPAR phosphorylation and dephosphorylation during synaptic plasticity. All the model outputs can be related to the phosphorylation and dephosphorylation of AMPARs. However, as the outputs of the models differ from each other, we also follow up the concentrations of active CaMKII and PP1, pivotal phosphorylating and dephosphorylating enzymes, respectively, in all the models. To compare the selected deterministic models [2, 16–19], we run simulations with several setups. Details of the simulation setups are given in Section 2.3.

#### 3.1.1. Models Describing Synaptic Plasticity in a Hippocampal CA1 Neuron

**Figure 3:**
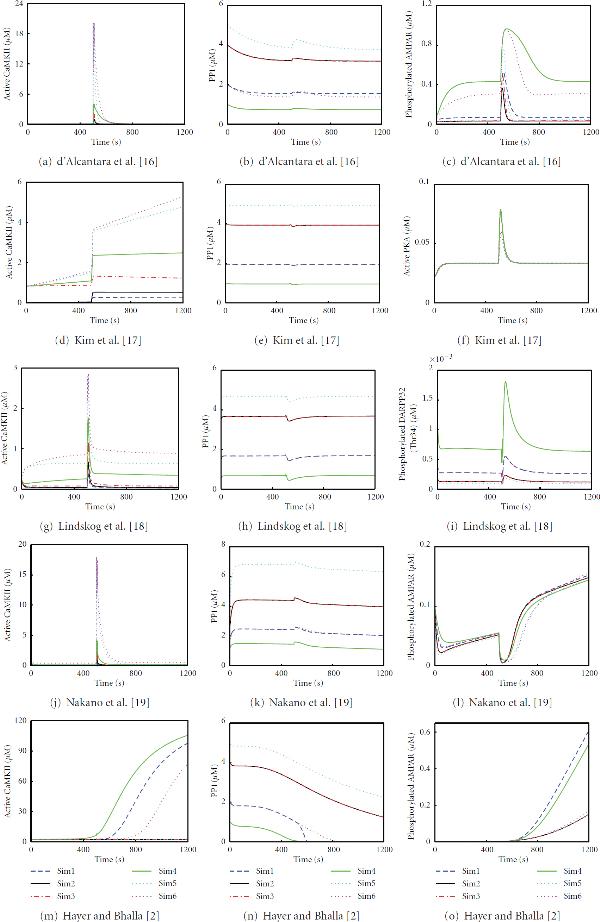
**Simulation results with different total concentrations of CaMKII and PP1**. First column presents active CaMKII, second column PP1 (most models have only one unbound form of PP1), and third column the selected output of each model. (a)–(o) show 1200 s of simulation time.

The concentrations of active CaMKII (see Figures 3(a) and 3(d)) in simulations of the hippocampal CA1 neuron models by d'Alcantara et al. [16] and Kim et al. [17] depend completely on the total concentration of CaMKII; the higher the total concentration of CaMKII, the higher the concentration of active CaMKII. In the case of the same total concentration of CaMKII (20 *μ*M in Sim5 and Sim6), the lower total concentration of PP1 produces higher concentration for active CaMKII. In this sense, simulations of the hippocampal CA1 neuron models by d'Alcantara et al. [16] and Kim et al. [17] show similar results for the concentrations of active CaMKII. Otherwise the model by Kim et al. [17] produces different responses for the concentration of active CaMKII compared to other models.

In the case of PP1 (see Figures 3(b) and 3(e)), the higher total concentration of PP1 produces higher concentration for PP1. Most models have only one unbound form of PP1 which concentration is plotted. Furthermore, the same total concentrations of PP1 (4 *μ*M in Sim2 and Sim3 and 2 *μ*M in Sim1 and Sim6) produce about the same concentrations for PP1.

The concentration of active PKA, which is the other output of the model by Kim et al. [17] in addition to the concentration of active CaMKII, varies very little due to the variation in total concentrations of CaMKII and PP1 (see Figure 3(f)). The simulations Sim1–Sim4, representing the ratios 0.25, 0.5, and 4 of the total concentrations of CaMKII and PP1, produce alike curves with peak concentrations of about 80 nM. In addition, the simulations Sim5 and Sim6, representing the ratios 4 and 10, produce slightly different peak concentrations (about 60 nM) but otherwise similar curves with each other and with other ratios as well. However, the model by d'Alcantara et al. [16] does not produce as straightforward results for the output of the model. Figure 3(c) shows the concentration of phosphorylated AMPAR simulated by the model of d'Alcantara et al. [16]. This model does not follow any pattern related to changes in the total concentrations of CaMKII and PP1 or the ratio of them.

#### 3.1.2. Models Describing Synaptic Plasticity in a Striatal Medium Spiny Neuron

The concentrations of active CaMKII and PP1 in simulations of the striatal medium spiny neuron models by Lindskog et al. [18] and Nakano et al. [19] follow similar behavior as the hippocampal CA1 neuron models (see Figures 3(g), 3(h), 3(j), and 3(k)). However, the actual concentrations vary even though the actual form of the curves can be similar. In this sense, simulations of the striatal medium spiny neuron models by Lindskog et al. [18] and Nakano et al. [19] show similar results for the concentrations of active CaMKII and PP1.

With the model by Lindskog et al. [18], the concentration of phosphorylated DARPP32 on threonine (Thr) 34 is plotted in Figure 3(i). Basically, this model output depends on the total concentration of PP1. If two simulations have the same total concentration of PP1, the concentrations of phosphorylated DARPP32 are the same. Furthermore, the lower the total concentration of PP1, the higher the concentration of phosphorylated DARPP32. However, the total concentrations of PP1 and CaMKII do not have a role for the concentration of phosphorylated DARPP32 on Thr75, thus it is about the same in all simulations (not shown). With the model by Nakano et al. [19], the concentration of phosphorylated AMPAR depends on the total concentration of PP1 before the input is given at 500 s (see Figure 3(l)). The lower the total concentration of PP1, the higher the concentration of phosphorylated AMPAR. However, after the input is given, the concentration of phosphorylated AMPAR does not follow any pattern related to changes in the total concentrations of CaMKII and PP1, or the ratio of them.

When simulating the model by Nakano et al. [19], we find out that the concentrations of active CaMKII and PP1 can reach higher than the total concentrations meaning they also appear elsewhere in the model. We have not found the reason for this even though we have marked all the initial concentrations related to them as zero. The problem is in the original model and not in the numerical integration. There is no easy way of debugging the code in Kinetikit either using the graphical user interface or modifying directly the model file. 

#### 3.1.3. Generic Neuron Model Describing Synaptic Plasticity

The concentration of active CaMKII from the model by Hayer and Bhalla [2] follows the total concentration of PP1 instead of the total concentration of CaMKII as in the other models (see Figure 3(m)). The lower the total concentration of PP1, the higher the concentration of active CaMKII. In the simulations Sim2 and Sim3, where the total concentration of PP1 is the same, the concentrations of active CaMKII stay on the same level. Earlier experimental results [20] have shown that CaMKII in the postsynaptic density can act as a stable switch, even in the presence of considerable phosphatase activity. Mullasseril et al. [20] justify the stability to be structural: CaMKII and PP1, both of which are in the postsynaptic density, are held in such a position that PP1 simply cannot reach the amino acid residue of CaMKII it is destined to dephosphorylate. This could be the experimental reasoning for the case in Figure 3(m), where the concentration of active CaMKII can rise high even though the total concentration of PP1 is considerably higher in respect to the total concentration of CaMKII (Sim1).

The concentration of PP1 follows similar behavior as the other models (see Figure 3(n)). The only exception is with Sim1, where the concentration of PP1 suddenly drops and does not behave similarly as in Sim6, as with the other models. The concentration of phosphorylated AMPAR does not follow any pattern related to changes in the total concentrations of CaMKII and PP1, or the ratio of them (see Figure 3(o)).

When simulating the model by Hayer and Bhalla [2], we set the total concentrations of CaMKII and PP1 only in the postsynaptic density. However, Hayer and Bhalla [2] also model diffusion of molecules between different compartments, being here between postsynaptic density and other compartments. Thus, the concentrations of active CaMKII and PP1 in the postsynaptic density can reach higher than the used total concentrations in the postsynaptic density.

#### 3.1.4. Comparison of Models

For all the models, the peak concentrations of active CaMKII and PP1 are tabulated together with the concentrations at the end point 2000 s in Table 5. Furthermore, percentages from the maximum peak concentration are given separately for each model. The peak concentrations of active CaMKII vary the most in different models. Especially in Sim1, the percentage of the model by Hayer and Bhalla [2] is the opposite compared to the percentage of the other models. As a surprise, the models by d'Alcantara et al. [16] and Nakano et al. [19] produce similar peak concentrations for active CaMKII even though they are made for neurons in different brain areas, the structures of the models are different, and Nakano et al. [19] do not report using the model by d'Alcantara et al. [16] as their base. The same can be concluded for the models by Lindskog et al. [18] and Kim et al. [17], but this can be explained by Kim et al. [17] using the model by Lindskog et al. [18] as their base. The end point concentrations of active CaMKII with the models by Hayer and Bhalla [2] and Kim et al. [17] are much higher than with the other three models. The peak and end point concentrations of PP1 are quite similar in all the models. The only exception is basically the model by Hayer and Bhalla [2] that produces much lower end point concentrations.

**Table 5:** *Concentrations of active CaMKII and PP1 in different simulations.* For all the models, the peak concentrations of active CaMKII and PP1 ([CaMKII]_peak_, [PP1]_peak_) are tabulated together with the concentrations at the end point 2000 s ([CaMKII]_end_, [PP1]_end_) in units of *μ*M. Furthermore, percentages from the maximum peak concentration are given separately for each model.

Sim ID	Model				
Sim1	d'Alcantara et al. [16]	0.4999 (3%)	0.0023	1.6276 (38%)	1.5507
	Kim et al. [17]	0.2912 (4%)^a^	0.2912	1.9634 (40%)	1.9440
	Lindskog et al. [18]	0.3617 (13%)	0.0251	1.7157 (36%)	1.6896
	Nakano et al. [19]	0.6707 (4%)	0.0173	2.5799 (37%)	1.7584
	Hayer and Bhalla [2]	117.9152 (99%)^a^	117.9152	2.0009 (40%)^a^	0.0002

Sim2	d'Alcantara et al. [16]	0.9998 (5%)	0.0030	3.3444 (78%)	3.1915
	Kim et al. [17]	0.5326 (8%)^a^	0.5174	3.9530 (80%)	3.9313
	Lindskog et al. [18]	0.6760 (24%)	0.0370	3.7155 (79%)	3.6893
	Nakano et al. [19]	1.1637 (6%)	0.0154	4.5621 (65%)	3.6606
	Hayer and Bhalla [2]	6.9027 (6%)^a^	6.9027	4.0004 (80%)^a^	0.1017

Sim3	d'Alcantara et al. [16]	1.9996 (10%)	0.0052	3.3480 (78%)	3.1744
	Kim et al. [17]	1.3295 (19%)^a^	1.1511	3.9524 (80%)	3.9295
	Lindskog et al. [18]	1.1739 (41%)	0.0737	3.7155 (79%)	3.6892
	Nakano et al. [19]	2.1318 (12%)	0.0286	4.5622 (65%)	3.6603
	Hayer and Bhalla [2]	6.9032 (6%)^a^	6.9032	4.0000 (80%)^a^	0.1017

Sim4	d'Alcantara et al. [16]	3.9996 (20%)	0.0341	0.8032 (19%)	0.7410
	Kim et al. [17]	2.5904 (37%)^a^	2.5904	0.9749 (20%)	0.9568
	Lindskog et al. [18]	1.7671 (61%)	0.3229	0.7160 (15%)	0.6890
	Nakano et al. [19]	4.0736 (23%)	0.2068	1.5902 (23%)	0.8950
	Hayer and Bhalla [2]	119.3471 (100%)^a^	119.3471	1.0006 (20%)^a^	0.0001

Sim5	d'Alcantara et al. [16]	19.9882 (100%)	0.0320	4.2702 (100%)	3.7504
	Kim et al. [17]	5.9959 (85%)^a^	5.9959	4.9522 (100%)	4.9102
	Lindskog et al. [18]	2.8245 (98%)	0.6318	4.7143 (100%)	4.6870
	Nakano et al. [19]	18.0171 (100%)	0.1727	6.9654 (100%)	6.0135
	Hayer and Bhalla [2]	20.0000 (17%)^a^	2.2876	5.0000 (100%)^a^	1.2884

Sim6	d'Alcantara et al. [16]	19.9933 (100%)	0.0686	1.6728 (39%)	1.3771
	Kim et al. [17]	7.0283 (100%)^a^	7.0283	1.9663 (40%)	1.9331
	Lindskog et al. [18]	2.8756 (100%)	0.8772	1.7143 (36%)	1.6862
	Nakano et al. [19]	18.0415 (100%)	0.5670	2.5900 (37%)	1.7571
	Hayer and Bhalla [2]	115.3967 (97%)^a^	115.3967	2.0000 (40%)^a^	0.0003

^a^ The maximum value is given here because the data in Figure 3 does not show a peak.

### 3.2. User Experiences

The model by d'Alcantara et al. [16] is easy to implement in MATLAB, since all the necessary information is given in the original publication; the model can also be found in BioModels database (http://www.biomodels.net/, [12]) in Systems Biology Markup Language (SBML, http://sbml.org/) format.

The models by Kim et al. [17] and Lindskog et al. [18] can be found in ModelDB (http://senselab.med.yale.edu/modeldb/, [26, 27]) in XPPAUT format (http://www.math.pitt.edu/~bard/xpp/xpp.html, [21]). The codes are properly commented and divided into several subsections. Thus, it is easy to find the value one wants to change to modify the model. However, the use of XPPAUT requires some practise, because the menu is not intuitive for first-time users.

The model by Nakano et al. [19] can be found in ModelDB in GENESIS/Kinetikit format (http://www.genesis-sim.org/GENESIS/, http://www.ncbs.res.in/node/350/, [22, 23]). In the database, the authors provide scripts for reproducing the figures in the original publication. As supplementary information of the original publication, they provide tables of initial concentrations and enzymatic and binding reactions. These tables are of great value when getting to know the model because the original model files are not commented and the language used for describing the model is not intuitive. Kinetikit provides a possibility to export an equation file which is also helpful. Unfortunately, the file lacks the sum equations of molecular species. This is particularly inconvenient with the model by Nakano et al. [19] because many of the active enzymes, including CaMKII and PP1, are sums of many different forms of theirs. This causes the problem with excess CaMKII and PP1 mentioned in Section 3.1.2. Kinetikit can be used either from command line or from graphical user interface which is useful since many times different users prefer different ways of simulation.

The model by Hayer and Bhalla [2] can be found in database of quantitative cellular Signaling (DOQCS) (http://doqcs.ncbs.res.in/, [24]) in several formats from which we have used the MATLAB format. However, the MATLAB implementation of the model is hard to modify, since, for example, rate constants and reaction rates are not given as vectors, and stoichiometric constants are not given as a matrix. Thus, if the user wants to change one parameter value, one is required to change the value everywhere it is used in the code. This is time consuming. Despite this problem, we prefer the MATLAB format over the Kinetikit format because modifications required in this study are easily and reliably done in MATLAB.

## 4. Discussion and Conclusions

In this study, we provide the first computational comparison of models for synaptic plasticity. Five different models [2, 16–19] describing the phenomena of LTP and LTD were selected for comparison, mainly due to their availability in model databases. The models were evaluated according to the model outcomes and the obtained user experiences to modify and simulate the models in certain simulation tools. We carefully examined the input-output relationship of the models. For this examination, we ran for each model six different simulations that were in advance known to produce physiologically realistic results. Our study revealed that when using exactly the same input, models describing the LTP/LTD phenomenon in the very same neuron type produced different responses. This may partly be explained by the fact that some models had been constructed to ask relatively specific questions using a certain simulation tool. On the other hand, the models by d'Alcantara et al. [16] and Nakano et al. [19] produced similar kind of results even though they had been built for neurons in different brain areas, and Nakano et al. [19] did not report using the model by d'Alcantara et al. [16] as their base. Almost the same can be concluded to the hippocampal CA1 neuron model by Lindskog et al. [18] and the striatal medium spiny neuron model by Kim et al. [17], but this can be explained by Kim et al. [17] using the model by Lindskog et al. [18] as their base.

In our previous study, we sought to classify and analyze the features of all existing LTP and LTD models without performing time-consuming computational simulations [5]. After running the simulations in this study, we discovered that it is extremely difficult to compare the models to each other, since objective methods, such as Bayesian methods, are not applicable. With this study, we try to motivate the research community to make a step forward to find a general setup how to compare models for synaptic plasticity.

We propose that all models should (1) be formulated using common description language, (2) have adequate metadata related to model and experimental data used, (3) explain set of features describing the overall behavior of the modeled system, and (4) be compared to previous models. In other words, all new models should be constructed according to clearly defined general rules. The four points presented above can be called the minimum criteria that the models need to meet as also explained in different BioModels projects (see, e.g., [11, 12]) and by Manninen et al [5]. Similar ideas about combining unified experimental findings that the models should capture are presented by Lisman and Raghavachari [25]. Several model databases are also available to store models and metadata for future use, for example, the BioModels database [12], ModelDB [26, 27], and DOQCS [24]. In addition, an international initiative, NeuroML (http://www.neuroml.org/), to develop language for describing detailed models of neural systems [28] and a model description practice for realistic neuronal network models [29] have been presented. The NeuroML initiative, however, still requires solutions to properly link signal transduction pathways and subcellular phenomena with cellular phenomena. This is a clear problem in the case of LTP/LTD phenomenon which requires several scales to be represented in the model. Regardless of this development, many models are neither constructed nor validated based on previous models because most computational neuroscientists use the so-called rebuild-from-scratch (de novo) methodology in model formation, as described by Cannon et al. [30].

The field of computational neuroscience is moving forward with every hypothesis tested and verified with simulations. Despite the fact that many models are not well documented and reproducible, there exist several well-established models that are frequently used (for short-term plasticity, see, e.g. [31]). Similar models are clearly needed also for long-term plasticity in different brain areas [32]. The purpose of our study is to advance the field and not as such to judge the previous studies. We, here, strongly propose that evaluators of scientific publications should require testing the model in the context of minimum criteria to see that the new model behaves as it should. In the best case, this would enable truly incremental science. In addition, the establishment of compulsory policies from publishers would partly solve the difficulties in data sharing and deposit of data files into public databases and repositories [33, 34] as well as the lack of experimental metadata in neuroscience [35].
